# Hemodynamic Risk Assessment by Thermodilution and Direct Fick Measurement of Cardiac Output in Pulmonary Hypertension

**DOI:** 10.1016/j.chpulm.2024.100059

**Published:** 2024-04-26

**Authors:** Adam J. Brownstein, Christopher B. Cooper, Sonia Jasuja, Alexander E. Sherman, Rajan Saggar, Richard N. Channick

**Affiliations:** Division of Pulmonary and Critical Care Medicine, University of California, Los Angeles, Los Angeles, CA

**Keywords:** direct Fick cardiac output, pulmonary hypertension, thermodilution cardiac output

## Abstract

**Background:**

Accurate measurement of cardiac output (CO) is critical in the evaluation and monitoring of pulmonary hypertension (PH). We assessed the accuracy of thermodilution (TD) CO vs direct Fick (DF) CO among patients with PH and evaluated whether the method of CO measurement affected diagnosis or risk assessment.

**Research Question:**

Does using Thermodilution CO as compared to Direct Fick CO alter hemodynamic risk status in PH?

**Study Design and Methods:**

We included patients who had undergone a right heart catheterization with both TD CO and DF CO measurements at University of California, Los Angeles between January 2021 and January 2023. Based on the cardiac index, patients were classified into low-, intermediate-, or high-risk hemodynamic status according to the European Society of Cardiology/European Respiratory Society guidelines.

**Results:**

The analysis included 116 patients with PH. Of the patients, 55% were on PH therapy at the time of catheterization. The median age was 59 years (25th-75th percentile, 50-69), and 63% were female. The median TD CO and DF CO were 4.6 L/min (25th-75th percentile, 3.6-6.0) and 5.3 L/min (25th-75th percentile, 4.2-7.0) (*P* = .007), respectively. Bland-Altman analysis revealed a mean bias of −0.64 L/min. Median DF pulmonary vascular resistance and TD pulmonary vascular resistance were 4.7 Wood units (25th-75th percentile, 2.7-6.6) and 5.6 Wood units (25th-75th percentile, 3.0-8.0), respectively. Among patients with a low TD cardiac index, almost 40% had a preserved DF cardiac index. There was 78% agreement between DF and TD hemodynamic risk status. Using TD over DF reclassified 8% of patients with precapillary PH (n = 101) from low-risk into intermediate- or high-risk hemodynamic status. TD had a sensitivity of 97% for appropriately risk stratifying patients into intermediate-/high-risk status but a specificity of 73%. Overall, there was a strong correlation between DF CO and TD CO (concordance correlation coefficient, 0.81; 25th-75th percentile, 0.74-0.86).

**Interpretation:**

Hemodynamic risk status was concordant between TD and DF measurements in almost 80% of patients. Oxygen consumption measurement should be considered if available on index right heart catheterization in patients with PH to aid in hemodynamic risk stratification or in whom strict pulmonary vascular resistance calculations are required.

**Take-home Points**

**Study Question:** Does hemodynamic risk assessment in precapillary pulmonary hypertension differ when using direct Fick (DF) compared with thermodilution (TD) cardiac output measurements?

**Results:** Hemodynamic risk status was concordant between TD and DF measurements in most patients (80%), but TD tended to underestimate cardiac output compared with DF and therefore led to identification of more patients with intermediate-risk hemodynamic status than DF.

**Interpretation:** Compared with DF, TD accurately identified hemodynamic status for most patients with pulmonary hypertension; however, oxygen consumption measurement with assurance of steady-state resting conditions should be considered if available on index right heart catheterization for DF cardiac output calculation to aid in appropriate hemodynamic pulmonary hypertension categorization and risk stratification, especially if there is discordance between TD-derived measurements and other variables used to guide risk assessment.

The appropriate diagnosis, categorization, and risk stratification of pulmonary hypertension (PH) rely on an accurate assessment of hemodynamics, including cardiac output (CO) and pulmonary vascular resistance (PVR).^1^^,^^2^ The 2022 European Society of Cardiology (ESC)/European Respiratory Society (ERS) guidelines^2^ recommend measuring CO by either the criterion standard direct Fick (DF) method or via thermodilution (TD), except in the presence of intracardiac shunts because TD is not accurate when these are present. The TD method, which is widely available in cardiac catheterization laboratories,^3^ involves the use of a pulmonary artery (PA) catheter that has a sensor that can detect the change in blood temperature on injection of a known volume of fluid with a known temperature.^4^ On the other hand, the DF method involves direct measurement of pulmonary oxygen consumption ($\dot{V}o_{2}$) using a Douglas bag^5^ or metabolic cart coupled with calculation of an arteriovenous oxygen concentration difference. Although the DF method is considered the gold standard approach, it is not widely available; thus, physicians must rely on TD in most scenarios. Nevertheless, there are limited data to determine if TD is adequate in the risk stratification of patients with PH compared with the DF method.

Studies have questioned the accuracy of TD CO compared with DF CO^6^^,^^7^; however, the primary indication for cardiac catheterization in these analyses was for the evaluation of intrinsic cardiac pathology (eg, valvular or ischemic disease). Given the importance of accurately diagnosing and risk stratifying patients with PH, we sought to assess the accuracy of TD CO vs DF CO among a population of patients with PH and evaluate whether using TD over DF would alter clinical management.

## Study Design and Methods

### Patient Selection

We included all patients who had undergone right heart catheterization (RHC) with both TD CO and DF CO measurements at the University of California, Los Angeles (UCLA) Medical Center cardiac catheterization laboratory between January 2021 and January 2023 as part of a new evaluation for PH or for monitoring of hemodynamics while on PH therapy. Patients were excluded from the final analysis if they had echocardiographic evidence of an intracardiac shunt. This study was approved by the UCLA Institutional Review Board (No. 12-000738). Demographics, baseline clinical characteristics, hemodynamics, and most recent transthoracic echocardiogram and spirometry data were collected via chart review.

### Definitions

PH was defined as mean pulmonary artery pressure (MPAP) > 20 mm Hg, and precapillary PH was defined as MPAP > 20 mm Hg, pulmonary arterial wedge pressure (PAWP) ≤ 15 mm Hg, and PVR ≥ 3 Wood units (WU) according to the Proceedings of the 6th World Symposium on Pulmonary Hypertension.^1^ Based on hemodynamic measurements and clinical assessment by expert physicians in our PH clinic, patients were classified into group I to V PH, according to published guidelines.^1^

Patients were stratified into preserved vs low cardiac index (CI) groups according to published guidelines.^2^ CI was considered low if < 2.5 L/min/m^2^ and preserved if ≥ 2.5 L/min/m^2^. TD CI and DF CI were classified as concordant if both were < 2.5 L/min/m^2^ or ≥ 2.5 L/min/m^2^. TD and DF PVR were classified as concordant if both were ≥ 3 WU or < 3 WU and discordant if not. Hemodynamic risk was determined to be low, intermediate, or high for each patient according to the ESC/ERS guidelines.^2^

### Measurements

Beginning in January 2021, regular attainment of resting $\dot{V}o_{2}$ directly preceding RHC in the Ronald Reagan UCLA Medical Center cardiac catheterization laboratory was implemented for patients with diagnosed or suspected PH. Resting $\dot{V}o_{2}$ was measured in the sitting position using the Vyntus CPX Metabolic cart (Vyaire) until a steady state was achieved. The system was calibrated prior to each $\dot{V}o_{2}$ measurement to ensure accuracy. Immediately after attainment of $\dot{V}o_{2}$, patients were placed in the supine position and hemodynamics were obtained using a 7 F balloon-tipped PA catheter with a TD port and PA temperature sensor. Vital signs were collected and monitored throughout the duration of RHC. Hemodynamics, including right atrial pressure, right ventricular pressure, PA pressure, and PAWP were recorded at end-expiration. After the aforementioned measurements were obtained, blood was drawn from the distal port for measurement of mixed venous oxygen saturation from the PA. Thereafter, at least three TD measurements were performed to obtain < 10% variability between measurements^8^ with injection of 10 mL of saline into the proximal port. Values were then averaged for each patient. DF CO was calculated according to the following equation: [measured $\dot{V}o_{2}$]/[(13.6 × hemoglobin (g/L) × (arterial oxygen saturation − mixed venous saturation)]. Arterial oxygen saturation was approximated with pulse oximetry. DF and TD PVR were calculated for each patient according to the following formula: (MPAP – PAWP)/CO. DF and TD CI were calculated using body surface area according to the formula of Du Bois and Du Bois.^9^ Indirect Fick CO was determined from estimated $\dot{V}o_{2}$ using three formulas: Dehmer et al,^10^ Bergstra et al,^11^ and LaFarge and Miettinen.^12^

### Statistics

Descriptive statistics are reported as median (quartile 1-quartile 3) for nonnormally distributed data and mean ± SD for normally distributed data. Intergroup comparisons for continuous variables were performed using either a paired *t* test or Wilcoxon rank sum test when appropriate. Categorical data were compared with χ^2^ or Fischer exact test when appropriate. The Bland-Altman method was used to assess the agreement of DF and TD CO, in which the difference between TD and DF CO was calculated for each patient and plotted against the corresponding average of DF and TD CO.^13^ Scatterplots were generated and concordance correlation coefficients were calculated between DF and TD CO measurements and between DF and TD CI in the stratified cohorts. Percent error (difference between TD and DF CO divided by DF CO and multiplied by 100) was also reported for DF and TD methods for the overall cohort and for the low and preserved CI subgroups. A McNemar χ^2^ test and calculation of Cohen kappa statistic were performed to assess the agreement between the two methods for risk stratifying patients with precapillary PH into low-, intermediate-, or high-risk hemodynamic status. Two-sided *P* < .05 was considered statistically significant. Statistical analysis was performed using R version 3.6.1 (the R Foundation for Statistical Computing).

## Results

**Figure 1:**
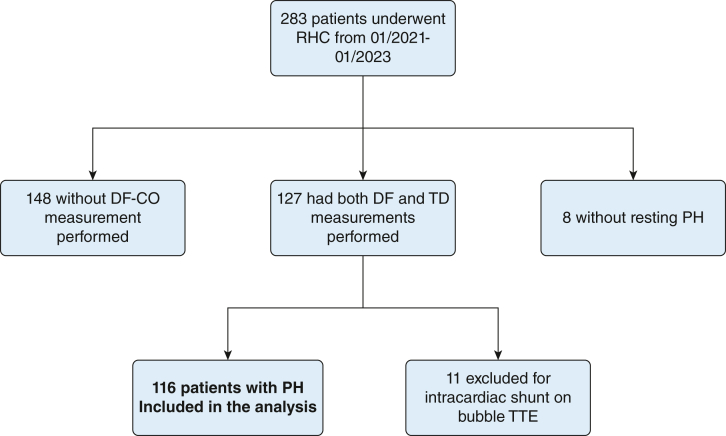
Consolidated Standards of Reporting Trials diagram showing patients at the University of California, Los Angeles undergoing RHC for investigation of pulmonary vascular disease between January 2021 and January 2023. After excluding patients without DF-CO, patients without PH, and patients with intracardiac shunt on TTE, 116 patients were included in our analysis. DF-CO = direct Fick measurements of cardiac output; DF = direct Fick; PH = pulmonary hypertension; RHC = right heart catheterization; TD = thermodilution; TTE = transthoracic echocardiogram.

A total of 283 patients (Fig 1) underwent RHC at the Ronald Reagan UCLA Medical Center from January 2021 to January 2023 as part of a hemodynamic evaluation for suspected PH or to monitor hemodynamic status while on PH therapy. One hundred twenty-seven patients with previously diagnosed or newly diagnosed PH underwent direct $\dot{V}o_{2}$ measurement immediately preceding RHC. Thirty of these patients underwent bubble contrast echocardiograms, of which 11 were positive for an intracardiac shunt. These patients were thus excluded, leaving a total of 116 patients for the primary analysis. Table 1 depicts the baseline characteristics of the study population. The median age was 59 years (quartile 1-quartile 3, 50-69), and 63% were female. A total of 55% were on PH therapy at the time of RHC and 75% were classified as group I PH. There was no statistically significant difference between the low and preserved CI groups in any of these variables. However, the low CI group had a significantly lower resting $\dot{V}o_{2}$ (254 ± 58 vs 299 ± 60 mL/min; *P* = .0002) and 6-min walking distance (275 ± 132 vs 391 ± 130 m; *P* = .001) compared with the preserved CI group and significantly higher MPAP (32 [quartile 1-quartile 3, 27-42] vs 43 [quartile 1-quartile 3, 33-53] mm Hg; *P* = .002), right atrial pressure (9 [quartile 1-quartile 3, 5-15] vs 6 [quartile 1-quartile 3, 4-9] mm Hg; *P* = .032), and PVR (8.6 [quartile 1-quartile 3, 5.8-13.1] vs 3.6 [quartile 1-quartile 3, 2.2-5.4] WU; *P* < .0001).

**Table 1:** Baseline Characteristics of the Study Population (N = 116)

Variable	Value
Age, y	59.5 (49.5-69.3)
Female	70 (60)
BMI, kg/m^2^	27.0 (22.1-30.9)
Any pulmonary vasodilator therapy	61 (52.5)
Single oral PH therapy	12 (10.3)
Combined oral/inhaled PH therapy	35 (30.2)
Parenteral PH therapy	14 (12.1)
Group I PH	83 (71.6)
Measured $\dot{V}o_{2}$, mL/min	284 ± 63
Direct Fick cardiac output, L/min	5.3 (4.2-7.0)
Thermodilution cardiac output, L/min	4.6 (3.6-6.0)
Direct Fick cardiac index, L/min/m^2^	2.9 (2.3-3.6)
Thermodilution cardiac index, L/min/m^2^	2.5 (2.0-3.1)
Mean pulmonary artery pressure, mm Hg	35 (28-48)
Pulmonary arterial wedge pressure, mm Hg	11 (8-14)
Right atrial pressure, mm Hg	7 (4-8)
Direct Fick pulmonary vascular resistance, Wood units	4.7 (2.7-6.6)
Thermodilution pulmonary vascular resistance, Wood units	5.6 (3.0-8.0)
Direct Fick stroke volume index, mL/m^2^	39.5 (29.0-50.8)
Thermodilution stroke volume index, mL/m^2^	35.6 (27.0-43.7)
Direct Fick pulmonary artery compliance, mL/mm Hg	2.2 (1.4-3.1)
Thermodilution pulmonary artery compliance, mL/mm Hg	1.9 (1.2-2.9)
Mixed venous oxygen saturation, %	65 (59-70)
6-min walk distance, m	357 ± 140

Values are mean ± SD, No. (%), or median (quartile 1-quartile 3). PH = pulmonary hypertension; $\dot{V}o_{2}$ = oxygen consumption.

Median TD CO and DF CO were 4.6 L/min (quartile 1-quartile 3, 3.6-6.0) and 5.3 L/min (quartile 1-quartile 3, 4.2-7.0) (*P* = .007), respectively. The median percent error between DF and TD CO was 13.5% (quartile 1-quartile 3, 1.1%-23.1%). Median TD CI and DF CI were 2.5 L/min/m^2^ (quartile 1-quartile 3, 2.0-3.1) and 2.9 L/min/m^2^ (quartile 1-quartile 3, 2.3-3.6), respectively. When stratified by preserved DF CI (n = 77) vs low DF CI (n = 39), the median percent error between DF and TD CI was 14.9% (quartile 1-quartile 3, 2.9%-27.2%) in the preserved CI group and 10.2% (quartile 1-quartile 3, −4.5% to 17.7%) in the low CI group (*P* = .03).

**Figure 2:**
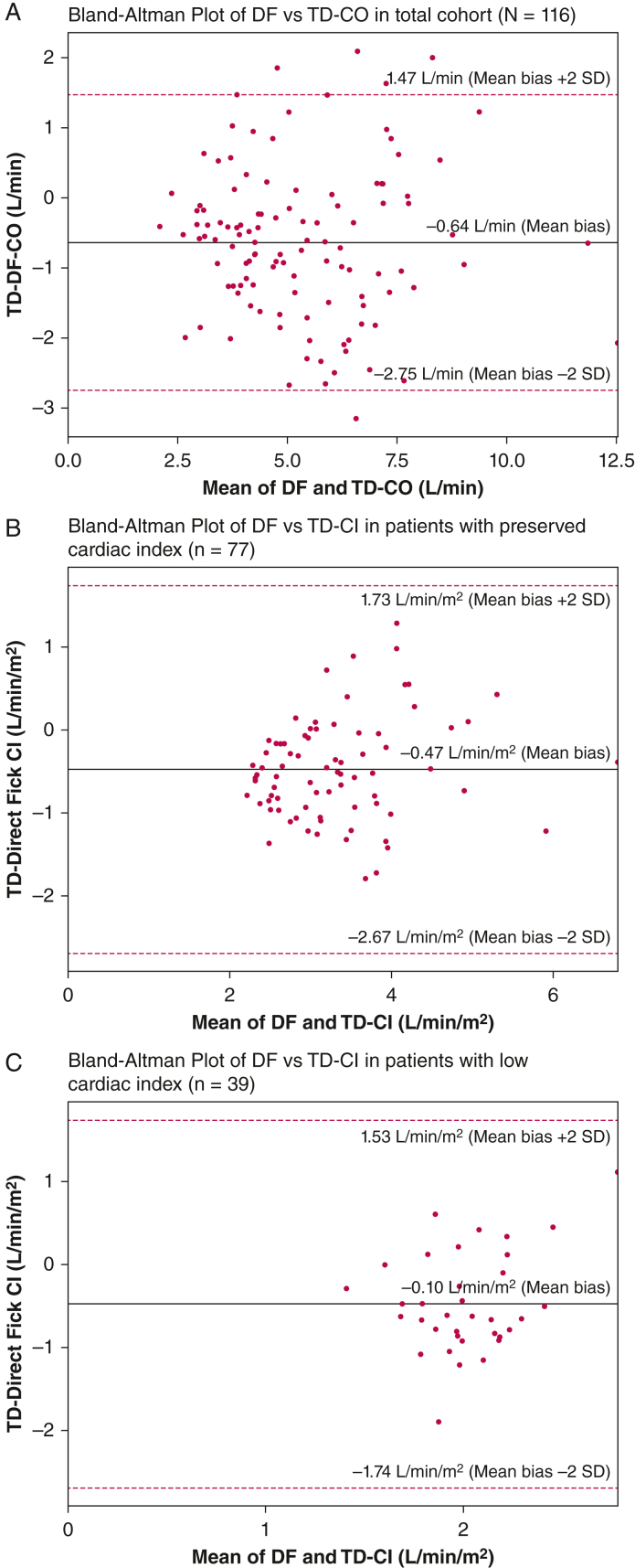
A-C, Agreement between DF and thermodilution methods using the Bland-Altman plot in the (A) overall cohort, (B) preserved DF-CI group, and (C) low DF-CI group. DF = direct Fick; DF-CO = Direct Fick cardiac output; TD-CI = Thermodilution cardiac index; TD-CO = Thermodilution cardiac output.

**Figure 3:**
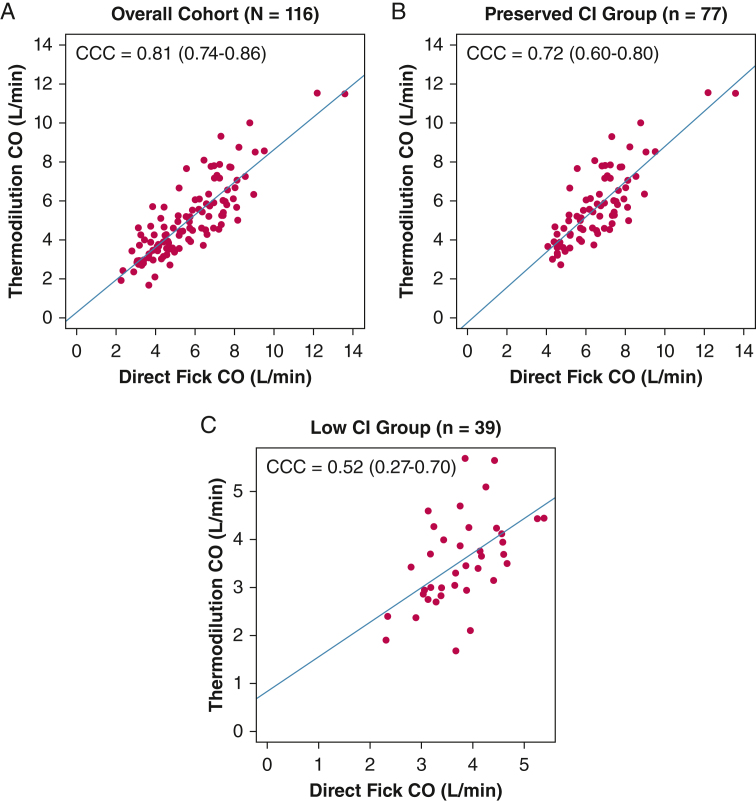
A-C, Relationship between direct Fick vs thermodilution methods in the (A) overall cohort, (B) preserved CI group, and (C) low CI group. CCC = concordance correlation coefficient; CI = cardiac index; CO = cardiac output.

The underestimation of CO by TD compared with DF was examined by the Bland-Altman method, which showed a mean bias (± 2 SDs) of −0.64 L/min (quartile 1-quartile 3, −2.75 to 1.47) (Fig 2, Table 2). Linear regression did not demonstrate a significant association between the mean of DF CO and TD CO with the difference between the two values (*P* = .73). When stratified by preserved vs low DF CI, the mean bias was −0.47 L/min/m^2^ (quartile 1-quartile 3, −2.67 to 1.73) for the preserved CI group and −0.10 L/min/m^2^ (quartile 1-quartile 3, −1.74 to 1.53) for the low CI group. The absolute difference in mean bias between the preserved and low CI groups was statistically significant (0.37 L/min/m^2^; quartile 1-quartile 3, 0.18-0.56; *P* = .0003). Figure 3 depicts plots of TD and DF CO in the overall cohort and TD and DF CI in the stratified cohorts. In the overall cohort, the concordance correlation coefficient between DF and TD CO was 0.81 (quartile 1-quartile 3, 0.74-0.86), whereas the concordance correlation coefficient was 0.72 (quartile 1-quartile 3, 0.60-0.80) in the preserved CI group and 0.52 (quartile 1-quartile 3, 0.27-0.70) in the low CI group.

**Table 2:** Accuracy of TD Method Compared With DF CO Method

Variable	No. of Patients	Mean Bias (± 2 SDs)	Median Percent Error (Q1-Q3)	Concordance Correlation Coefficient (95% CI)
Overall TD vs DF CO	116	−0.64 (−2.75 to 1.47)	13.5 (1.1 to 23.1)	0.81 (0.74-0.86)
DF CI ≥ 2.5 L/min/m^2^	77	−0.47 (−2.67 to 1.73)	14.9 (2.9 to 27.2)	0.72 (0.60-0.80)
DF CI < 2.5 L/min/m^2^	39	−0.10 (−1.74 to 1.53)	10.2 (−4.5 to 17.7)	0.52 (0.27-0.70)

The negative mean bias indicates the degree to which TD underestimated CO compared with DF. CI = cardiac index; CO = cardiac output; DF = direct Fick; Q = quartile; TD = thermodilution.

Agreement between indirect Fick CO and both DF CO and TD CO is shown in Table 3, Table 4, respectively. All three indirect Fick formulas tended to underestimate DF CO with a mean bias of −1.08 L/min (quartile 1-quartile 3, −3.44 to 1.28) for the Dehmer et al^10^ formula, −0.58 L/min (quartile 1-quartile 3, −2.91 to 1.74) for the Bergstra et al^11^ formula, and −3.04 L/min (quartile 1-quartile 3, −5.92 to −0.16) for the LaFarge and Miettinen^12^ equation. The Bergstra et al^11^ formula most closely approximated TD CO with a mean bias of 0.04 L/min with limits of agreement ranging from −1.99 to 2.06 L/min.

**Table 3:** Accuracy of Indirect Fick Methods Compared With Direct Fick Cardiac Output

Indirect Fick Method	No. of Patients	Mean Bias (± 2 SDs), L/min	Median Percent Error (Q1-Q3)	Concordance Correlation Coefficient (95% CI)
Dehmer et al^10^	127	−1.08 (−3.44 to 1.28)	19.3 (4.7 to 29.3)	0.64 (0.55-0.71)
LaFarge and Miettinen^12^	127	−3.04 (−5.92 to −0.16)	54.0 (46.4 to 59.6)	0.18 (0.14-0.23)
Bergstra et al^11^	127	−0.58 (−2.91 to 1.74)	9.2 (−5.3 to 20.9)	0.75 (0.67-0.81)

The negative mean bias indicates the degree to which the indirect Fick underestimated cardiac output compared with the direct Fick. Q = quartile.

**Table 4:** Accuracy of Indirect Fick Methods Compared With Thermodilution Cardiac Output^a^

Indirect Fick Method	No. of Patients	Mean Bias (± 2 SDs)	Median Percent Error (Q1-Q3)	Concordance Correlation Coefficient (95% CI)
Dehmer et al^10^	116	−0.47 (−2.54 to 1.61)	9.5 (−8.4 to 17.6)	0.77 (0.70-0.83)
LaFarge and Miettinen^12^	116	−2.44 (−5.09 to 0.20)	48.0 (40.5 to 53.9)	0.26 (0.19-0.30)
Bergstra et al^11^	116	0.04 (−1.99 to 2.06)	−1.9 (−19.9 to 9.4)	0.83 (0.77-0.88)

The negative mean bias indicates the degree to which the indirect Fick underestimated cardiac output compared with thermodilution. Q = quartile.

a Excluded patients with evidence of an intracardiac shunt on bubble echocardiogram.

**Figure 4:**
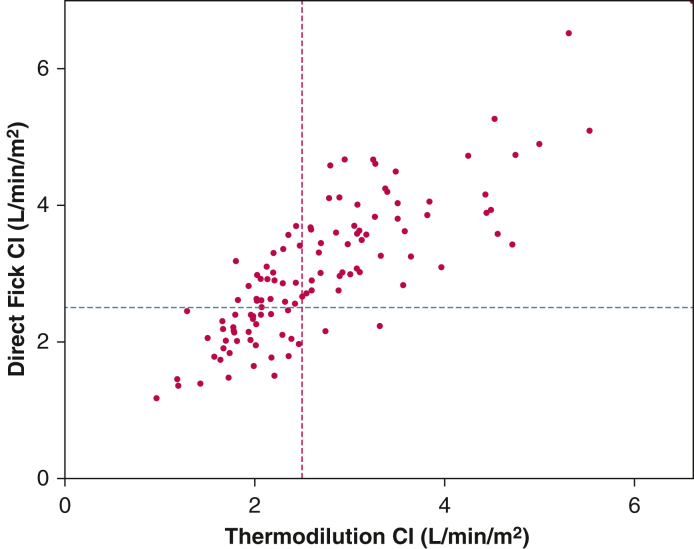
Performance of thermodilution in appropriately categorizing patients with pulmonary hypertension into low and preserved CI groups according to direct Fick. CI = cardiac index.

Median DF PVR and TD PVR were 4.7 WU (quartile 1-quartile 3, 2.7-6.6) and 5.6 WU (quartile 1-quartile 3, 3.0-8.0), respectively (*P* = .15). Twelve patients (11%) had discordant PVR calculations. In eight out of 12 patients, TD PVR was > 3 WU, whereas the DF PVR was < 3 WU. None of these patients had moderate or severe tricuspid regurgitation on pre-RHC transthoracic echocardiogram. A plot of DF vs TD CI is shown in Figure 4. TD and DF CI were concordant in 82% of patients. Only 3.6% of patients (n = 2) with preserved TD CI had a DF CI < 2.5 L/min/m^2^, whereas 38% of patients (n = 23) with TD CI < 2.5 L/min/m^2^ had a preserved DF CI. Using TD over DF reclassified eight patients (8%) with precapillary PH (n = 101) from low-risk into intermediate- or high-risk hemodynamic status based on published guidelines^2^ (Table 5). Overall, there was 78% agreement between DF and TD hemodynamic risk status. McNemar χ^2^ testing did not reveal a significant difference in hemodynamic risk status category using DF or TD methods (*P* = .08) with a Cohen kappa statistic of 0.651, indicating substantial agreement. We also assessed the diagnostic performance of the TD method for categorizing patients with precapillary PH into either intermediate-/high-risk status vs low-risk status compared with the DF method. TD had a sensitivity of 97% for risk stratifying patients into intermediate-/high-risk status and a negative predictive value of 92% for ruling out intermediate-/high-risk status. TD had a specificity of 73% for determining intermediate-/high-risk status, yielding a false-positive rate of 27%.

**Table 5:** Hemodynamic Risk Profile for Patients With Precapillary Pulmonary Hypertension (n = 101) by DF and TD Methods

Risk Status	DF High Risk	DF Intermediate Risk	DF Low Risk
TD high risk	44 (88)	7 (33)	0 (0)
TD intermediate risk	5 (10)	13 (62)	8 (27)
TD low risk	1 (2)	1 (5)	22 (73)

Values are No. (%). Risk status per the 2022 European Society of Cardiology/European Respiratory Society guidelines.^2^ DF = direct Fick; TD = thermodilution.

Univariable linear regression analysis was performed to determine if there was an association of variables of interest with TD CO error (absolute difference of TD and DF CO in the overall cohort). e-Table 1 illustrates that higher values of resting $\dot{V}o_{2}$ were significantly associated with a greater negative bias (ie, an underestimation of DF CO by TD). MPAP, PAWP, systolic BP, and presence of moderate or severe tricuspid regurgitation on transthoracic echocardiogram obtained a median of 82 days prior to RHC were not significantly associated with TD CO error.

## Discussion

This retrospective single-center study evaluated the agreement between DF and TD methods for calculating CO in a cohort of patients with PH and sought to determine the accuracy of TD for appropriately risk stratifying patients according to their hemodynamics on RHC. Our main findings are the following: (1) there was strong agreement between DF and TD CO measurements, (2) a Bland-Altman analysis revealed that TD tended to underestimate DF CO, (3) almost 40% of patients with a low TD CI had a preserved DF CI, and (4) hemodynamic risk status was concordant between TD and DF measurements in almost 80% of patients. Although the sensitivity of TD for appropriately identifying intermediate-/high-risk hemodynamic status according to DF is very high, using TD identified more intermediate-risk hemodynamic patients than DF.

Although TD and DF are presented in the ESC/ERS guidelines as comparable methods for assessing hemodynamics in the absence of cardiac shunts based on prior studies demonstrating their strong concordance,^14^, ^15^, ^16^ including patients with PH,^17^, ^18^, ^19^ the accuracy of TD has been questioned.^6^^,^^7^ In the largest known study published to date comparing TD and DF CO (N = 253) among patients undergoing cardiac catheterization for either hemodynamic, ischemic, or valvular assessment, Narang et al^7^ found a median percent error of 17.5% between the two methods.

Although an accurate determination of CI is critical in multiple clinical scenarios,^3^^,^^7^ few studies have evaluated whether using TD over DF could potentially alter clinical management in patients with PH. Therefore, we sought to examine if using TD over DF would change the hemodynamic risk profile of this patient population.

Because TD CO tended to be lower than DF CO, TD PVR tended to be higher. This discrepancy led to five excess diagnoses of precapillary PH. Similarly, in a cohort of 29 patients referred for invasive cardiopulmonary exercise testing, Hsu et al^20^ found a mean bias of −1.81 L/min and that using TD CO over DF CO led to an overdiagnosis of exercise-induced PH. Reliance on TD CO may also have important implications for patients in whom strict PVR targets are required (eg, those with portopulmonary hypertension). Specifically, the recently revised model for end-stage liver disease exception criteria states that patients with portopulmonary hypertension on the liver transplant waiting list receiving treatment for PH can be considered for exception points if the MPAP is < 35 mm Hg and PVR < 5 WU or if the MPAP is 35 to 45 mm Hg and PVR < 3 WU.^21^ Furthermore, because patients with portopulmonary hypertension tend to have higher baseline CO^22^ and TD bias increases as CO increases, the use of TD over DF in patients with portopulmonary hypertension may inappropriately label patients with hemodynamics that do not meet criteria for liver transplantation. However, these hemodynamic criteria were not derived using DF CO to calculate PVR.

In a study of 35 patients with PH, Hoeper et al^18^ found only a mean bias of 0.01 ± 1.1 L/min, whereas Yung et al^19^ found that the correlation coefficient between TD and DF CO was 0.89 with a mean bias of 0.19 L/min in a study of 39 patients with PH. In a cohort of 75 patients with PAH, Khirfan et al^17^ found an intraclass correlation coefficient of 0.88 between DF CI and TD CI with a mean bias of 0.01 L/min/m^2^. Sahay et al^23^ found a mean difference of −0.4 ± 1.3 L/min between TD and DF CO in 151 patients referred for RHC, whereas Duknic et al^24^ found a mean bias of −0.45 L/min (limits of agreement, −2.93 and 2.03 L/min) in 24 patients with PH. Our study of 116 patients with newly diagnosed or previously diagnosed PH undergoing follow-up hemodynamic assessment found that, although the correlation between the two methods was strong, TD tended to underestimate CO compared with DF. We stratified patients into preserved (≥ 2.5 L/min/m^2^) and low CI (< 2.5 L/min/m^2^) groups and found a significantly larger mean bias among patients with preserved CI. These findings are in agreement with prior studies demonstrating that mean bias is larger as CO increases.^7^^,^^20^ Similar to Hoeper et al,^18^ we found that the presence of moderate or severe tricuspid regurgitation on pre-RHC echocardiogram was not significantly associated with an increased absolute TD error.

In our risk stratification analysis, we observed a 78% agreement between DF and TD hemodynamic risk status according to ESC/ERS guidelines, which is slightly higher than that observed by Khirfan at al^17^ (72%). However, we found that 27% of patients with precapillary PH with low-risk hemodynamic status according to DF (n = 30) would be reclassified to intermediate-risk status if TD is used instead and that 33% of patients classified as having intermediate-risk hemodynamics by DF (n = 21) would be reclassified into high-risk status if TD is used. Our findings have important implications for patient care given the guideline recommendations to target low-risk status in patients with precapillary PH.^2^ Importantly, we also found that among patients with a low TD CI, almost two out of five had a preserved DF CI. Given the prognostic significance of CI in patients with PH and its importance for both risk stratification and determining the need for escalation of PH therapy,^25^, ^26^, ^27^, ^28^ an accurate measurement of CI is critical. Furthermore, CO is incorporated into calculations of stroke volume index and pulmonary arterial compliance, which are important prognostic markers in PH.^2^^,^^29^ Our results suggest that a reliance on TD over DF CI could potentially lead to an escalation of PH therapy that may not be warranted in a subset of patients with low TD CI.

In our univariable linear regression analysis, TD error was significantly higher among patients with a higher resting $\dot{V}o_{2}$. We examined the effect of resting $\dot{V}o_{2}$ on the calculated TD error using published equations for its estimation. Similar to prior analyses,^17^ we found that CO calculations according to the Dehmer et al^10^ and Bergstra et al^11^ formulas were closer in agreement to both DF and TD CO than the LaFarge and Miettinen^12^ equation. Based on our experience, and the experience of others, we therefore recommend against using the LaFarge and Miettinen^12^ equation for estimating $\dot{V}o_{2}$ in patients with PH.

There are several limitations to our study. First, arterial oxygen saturation was estimated using pulse oximetry as opposed to direct analysis from an arterial blood gas. Second, $\dot{V}o_{2}$ measurements were not done on patients requiring supplemental oxygen, which limits applicability of our results. Third, only approximately one-quarter of the cohort underwent contrast bubble echocardiogram to evaluate for patent foramen ovale, which could affect TD CO. However, if a patent foramen ovale with right to left shunting was present, one would expect a falsely higher TD CO, not a lower TD CO as we found. Fourth, $\dot{V}o_{2}$ was measured in the sitting position as opposed to the supine position. However, given that both TD measurements were performed and arterial venous oxygen content differences were determined in the supine position, we would not expect that measurement of $\dot{V}o_{2}$ in the sitting position would affect our findings. Additionally, one study demonstrated no significant difference in measured $\dot{V}o_{2}$ among women in the sitting vs supine position.^30^ Finally, given we found a positive association between TD CO error and the difference in measured vs predicted $\dot{V}o_{2}$, unsteady state conditions or technical issues could have led to an overestimation of resting $\dot{V}o_{2}$ and thus an overestimation of DF CO.

## Interpretation

We found that in a population of patients with newly diagnosed or previously diagnosed PH on therapy undergoing hemodynamic assessment, TD correlated well with DF for most patients, including among those with low CI, but tended to underestimate CO. This has two main clinical implications. First, more patients were diagnosed with precapillary PH if one solely relied on TD. Second, although we observed a high concordance in hemodynamic risk status between TD and DF methods, there were a higher number of patients with intermediate-risk hemodynamic status and fewer patients with low-risk hemodynamic status when using TD. Our results indicate that $\dot{V}o_{2}$ measurement with assurance of steady-state resting conditions should be considered if available on index RHC for DF CO calculation to aid in appropriate hemodynamic PH categorization and risk stratification, especially if there is discordance between TD-derived measurements and other variables used to guide risk assessment.

## Funding/Support

Research was supported by the National Center for Advancing Translational Science of the National Institutes of Health under the 10.13039/100016206UCLA Clinical and Translational Science Institute [Grant UL1TR001881].

## Financial/Nonfinancial Disclosures

The authors have reported to *CHEST Pulmonary* the following: A. J. B. was supported by the National Center for Advancing Translational Science of the National Institutes of Health under the UCLA Clinical and Translational Science Institute [Grant UL1TR001881] during the conduct of this study. C. B. C reports grants from the NIH/NHLBI, NIH Foundation, and COPD Foundation, during the conduct of the study; and personal fees from AstraZeneca, GlaxoSmithKline, Chiesi, NUVAIRA, MGC Diagnostics, Horizon Therapeutics, Respiree, Herbalife, Verona, and Cambridge University Press, outside the submitted work. R. S. receives consulting fees from United Therapeutics and Johnson & Johnson – Janssen Pharmaceuticals (Actelion Pharmaceuticals). R. C. receives consulting fees from United Therapeutics, Johnson & Johnson – Janssen Pharmaceuticals (Actelion Pharmaceuticals), Bayer HealthCare Pharmaceuticals Inc, Merck Sharp & Dohme LLC, and Penumbra Inc. None declared (S. J., A. E. S.).

## Supplementary Data

e-Table 1
