# Supplementary material for: Hemodynamic Risk Assessment by Thermodilution and Direct Fick Measurement of Cardiac Output in Pulmonary Hypertension
Source: CHEST Pulm. 2024 Apr 26;2(3):100059. doi: 10.1016/j.chpulm.2024.100059 (PMC13420477; doi:10.1016/j.chpulm.2024.100059)
Supplement: e-Table 1 [file mmc1.docx]

e-Table 1. Univariable linear regression analysis of the association of select variables with absolute TD error (difference between TD-CO and DF-CO)

| **Variable** | **Regression coefficient (95% CI)** | ***P*-value** |
| --- | --- | --- |
| Age (years) | -0.009 (-0.023 – 0.005) | 0.202 |
| Sex | 0.327 (-0.075 – 0.729) | 0.110 |
| Body mass index (kg/m^2^) | 0.017 (-0.009 – 0.044) | 0.196 |
| Resting V̇O_2_ (mL/min) | -0.008 (-0.010 – -0.005) | **<0.001** |
| mPAP (mm Hg) | 0.011 (-0.005 – 0.026) | 0.172 |
| PAWP (mm Hg) | 0.017 (-0.020 – 0.054) | 0.367 |
| Systolic blood pressure (mm Hg) | 0.001 (-0.008 – 0.009) | 0.847 |
| Presence of moderate or severe tricuspid regurgitation on pre-RHC echocardiogram | -0.167 (-0.590 – 0.255) | 0.434 |
