# Supplementary figures and images for: Hemodynamic Risk Assessment by Thermodilution and Direct Fick Measurement of Cardiac Output in Pulmonary Hypertension
Source: CHEST Pulm. 2024 Apr 26;2(3):100059. doi: 10.1016/j.chpulm.2024.100059 (PMC13420477; doi:10.1016/j.chpulm.2024.100059)

VO2

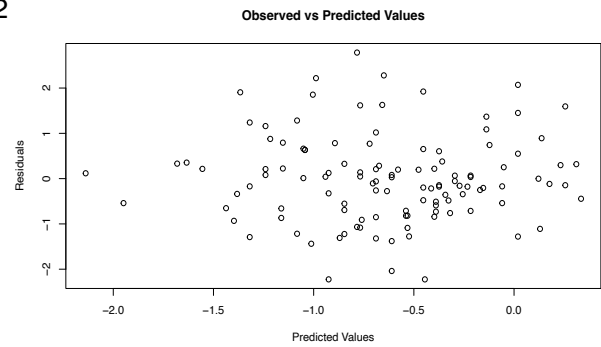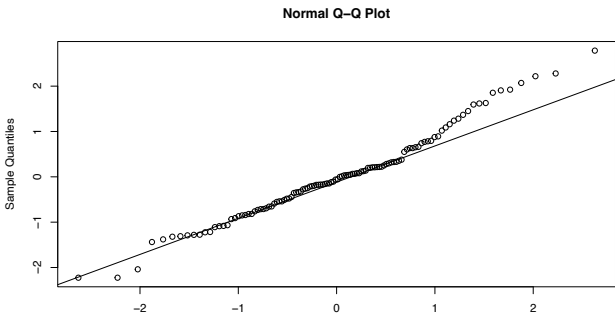

Age

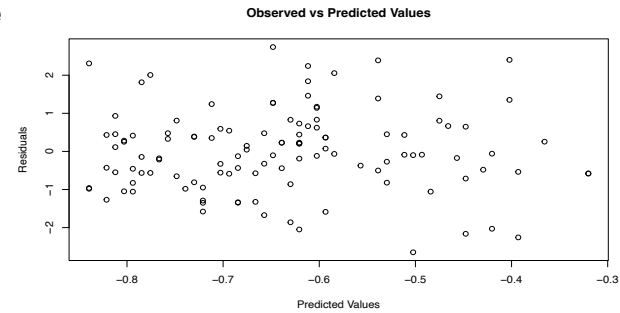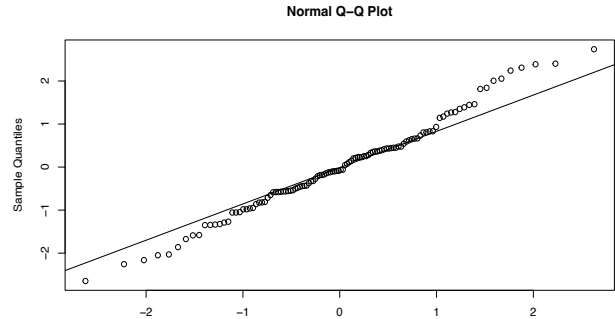

BMI

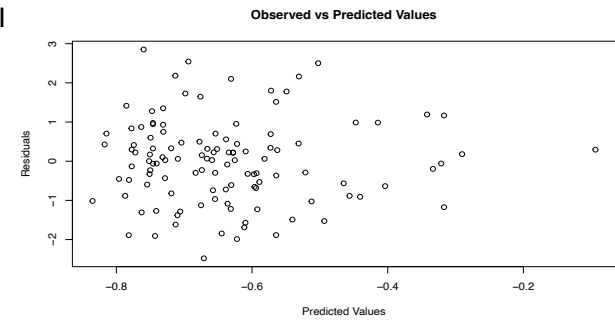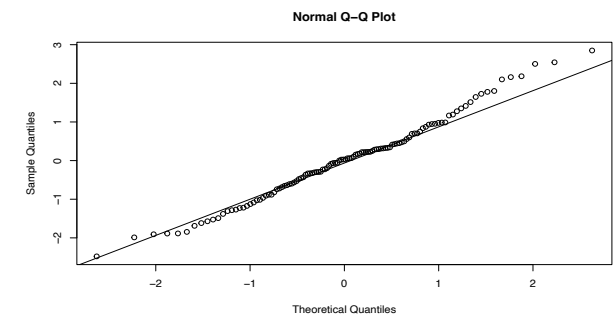

mPAP

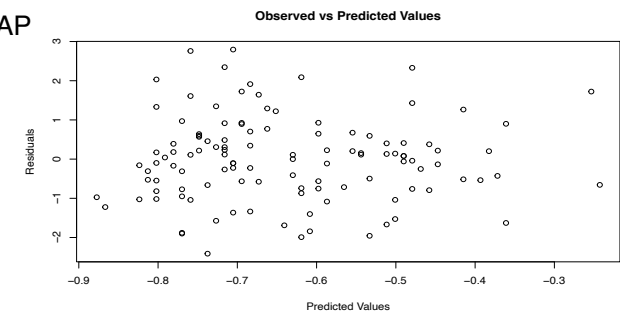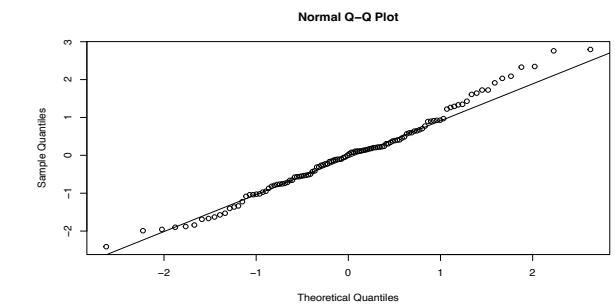

PAWP

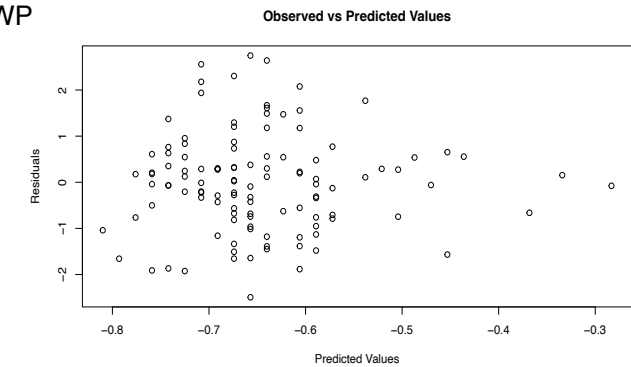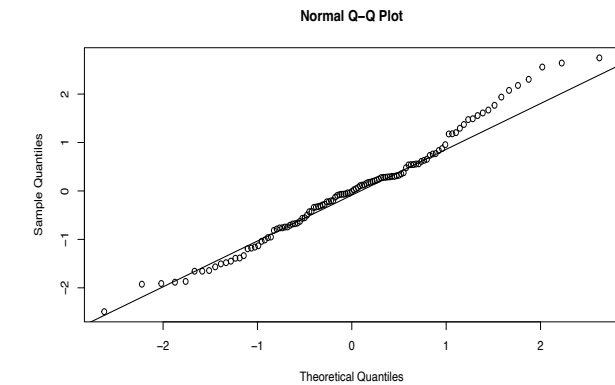

SBP

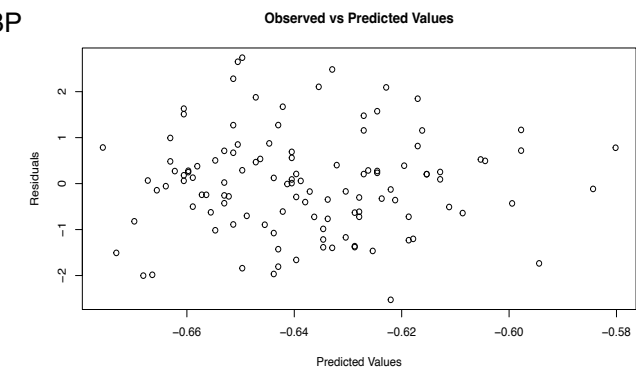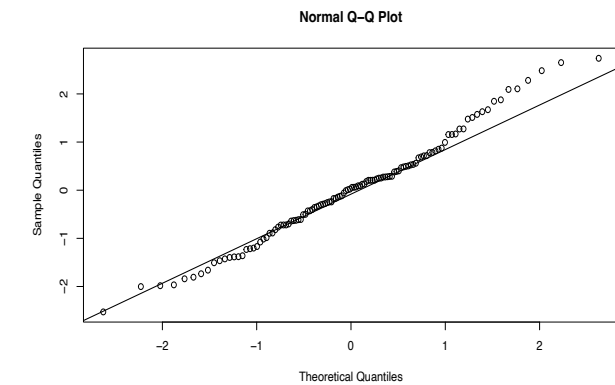

Supplement: Supplementary Data [file mmc2.pdf]
